# First observations of the bigfin squid *Magnapinna* sp. in the Colombian Southern Caribbean

**DOI:** 10.3897/BDJ.6.e24170

**Published:** 2018-05-04

**Authors:** Jurgen Guerrero-Kommritz, Jaime Cantera, Vladimir Puentes, Jorge Leon

**Affiliations:** 1 Fundabas, Bogota, Colombia; 2 Universidad del Valle, Cali, Colombia; 3 Anadarko company, Bogota, Colombia; 4 Anadarko, Bogota, Colombia

**Keywords:** Magnapinna, Southern Caribbean, Colombia, deep sea

## Abstract

Herein, first observations are reported of *Magnapinna* squids in the Colombian Southern Caribbean. Two specimens were observed by Remote Operated Vehicles (ROV) during exploratory drilling surveys for hydrocarbons at 1,883 and 2,294 m depth. These are the first observations of specimens of *Magnapinna* in the Southern Caribbean.

Resumen

La primera observación del calamar *Magnapinna* sp. en el caribe sur colombiano. Dos especímenes de calamares de aleta grande fueron observados con submarino de operación remota (ROV) durante un proyecto de perforación exploratoria de hidrocaburos a profundidades de 1,883 y de 2,294 m, respectivamente. Estas son las primeras observaciones de especímenes de *Magnapinna* en el Caribe Sur.

## Short communication

During routine inspection of exploratory wells by a Remotely Operated Vehicle (ROV) approximately 57 km off Arboletes and 67.1 km off Punta Broqueles (Moñitos, Cordoba), Colombian coast, two individuals of an uncommon deep-sea squid were observed in different locations. The first sighting (1:22 minutes) was in the Old Purple Angel Well (9°12'25,732"N; 76°49'55,091"W) on 14 February 2017 (08:24 am) at 1,884 m depth. The second sighting (1 minute) was at the Gorgon 1 well (9°25'59,282"N; 76°44'54,110"W) on 14 April 2017 (09:15 am) at 2,294.20 m depth.

According to the morphology of the specimens (Fig. 1a, b, c) they were identified as members of the genus *Magnapinna* Vecchione and Young, 1998 (Magnapinnidae Vecchione & Young, 1998) (Vecchione et al. 2001, Vecchione and Young 2016). Unfortunately, the species cannot be determined without close examination of some details of the arm and tentacle suckers and these details were not possible to see with the videos provided by the ROV in the two sightings. It was thus not possible to get confirmation of the species. This is a common problem with photographic identification of invertebrates. A review of the videos however, confirmed the family and genus (Michael Vecchione, Smithsonian Institute, Washington DC, personal communication).

The size of the specimens, estimated through comparison with the riser (set of tubes that connect the well with the drilling unit) is of approximately 200 mm mantle length; fin width 200 mm and a total length of ca. 2,300 mm. The individuals exhibited characteristics of fully developed animals: one specimen had its complete arm arrangement with all its filaments, the other specimen lacked the filament on one arm.

The poorly known deep-sea family Magnapinnidae, where only eleven specimens are known and deposited in collections worldwide, is presently understood to comprise five species: *Magnapinna pacifica* Vecchione & Young, 1998, *M. atlantica* Vecchione & Young, 2006, *M. talismani* (Fischer & Joubin, 1907), *Magnapinna* sp. B and *Magnapinna* sp. C (Vecchione and Young 1998, Vecchione and Young 2006, Vecchione and Young 2016). Vecchione et al. (2001) described the occurrence of a large, strange squid recorded only by submersible or ROV observations in the Atlantic, Indian and Pacific Oceans. Magnapinnid species are characterised by unique brachial morphology amongst all known cephalopods: the thicker regions of the arms and tentacles are often held at nearly right angles to the body axis and the long, slender portions of the arms and tentacles that trail the squid are nearly parallel to the body axis. Magnapinnids are likely found, at least in temperate to tropical latitudes, throughout the world's oceans at great depths within a few meters from the bottom. The deepest record is 4,735 m depth in the western Atlantic off the coast of Brazil (Vecchione et al. 2001, Vecchione and Young 2016). *Magnapinna* has been reported in the Great Caribbean and Gulf of Mexico by ROV sightings at the offshore exploratory well “Perdido” and during deep-sea research, respectively (Guerra et al. 2002). These are the first observations known for the family in the Southern Caribbean.

Until now, 49 species of cephalopods were known for the Colombian Caribbean (Guerrero-Kommritz 2015), most from shallow water (Diaz et al. 2000, Gracia et al. 2002). The only reports from deeper water are for *Semirossia* spp. and *Austrorossia* spp. from 500 and 790 m depth (Guerrero-Kommritz and Rodriguez-Bermudez 2017). This is the first report of a Magnapinnid (and of any deep-sea oegopsid) squid in the Colombian Caribbean.

## Figures and Tables

**Figure 1a.**
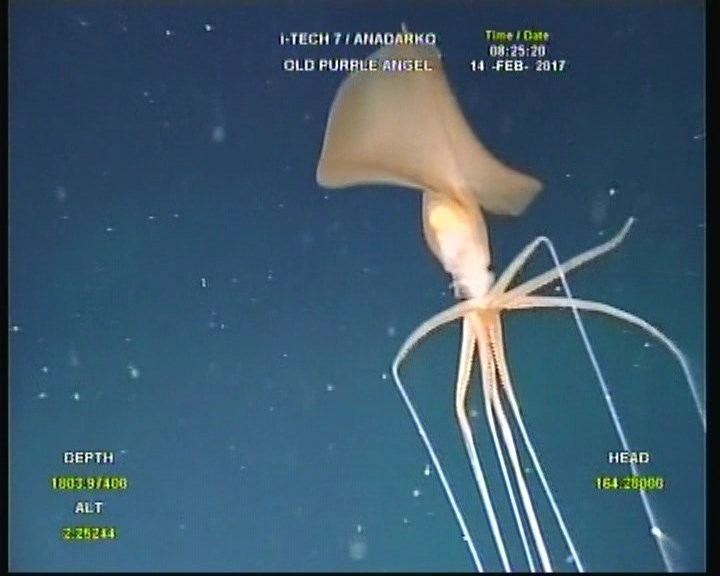
Specimen one drifting near Old Purple Angel well, showing the lack of one arm tip.

**Figure 1b.**
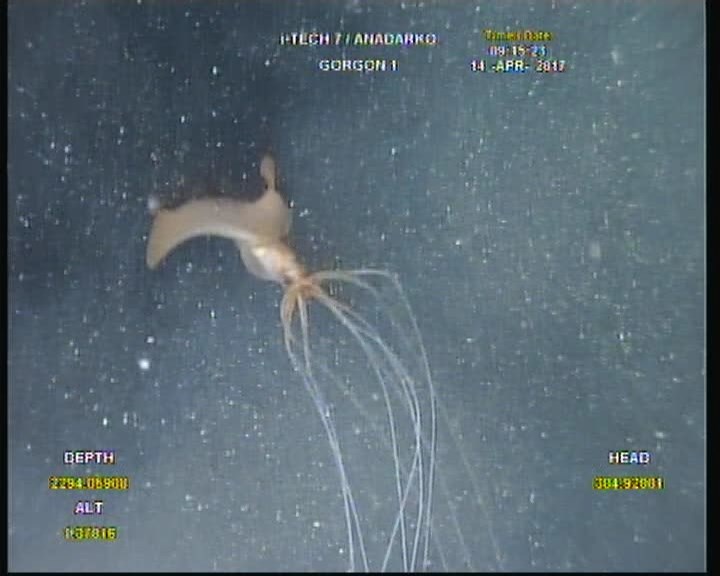
Specimen two drifting near Gorgon 1 well with complete arms.

**Figure 1c.**
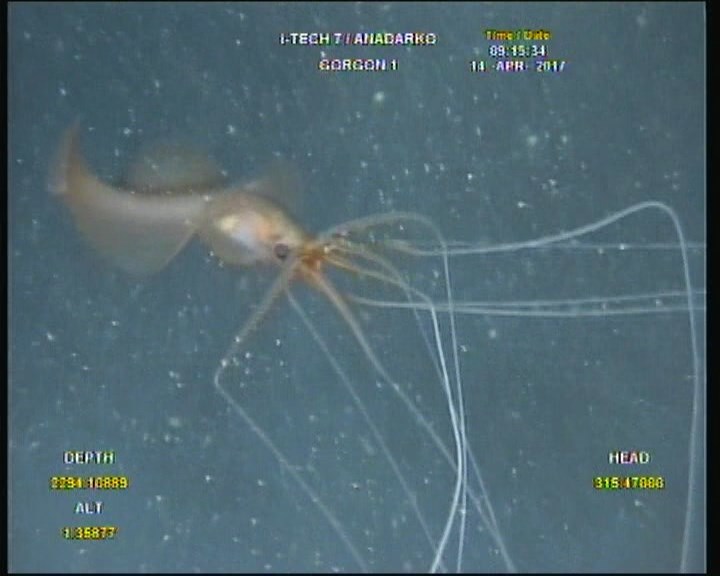
Specimen two near Gorgon 1 well swimming away.
